# Microplastic Pollution: Chemical Characterization and Impact on Wildlife

**DOI:** 10.3390/ijerph20031745

**Published:** 2023-01-18

**Authors:** Sumon Sarkar, Hanin Diab, Jonathan Thompson

**Affiliations:** School of Veterinary Medicine, Texas Tech University, 7671 Evans Dr., Amarillo, TX 79106, USA

**Keywords:** microplastics, fibers, one health

## Abstract

Microplastics are small pieces of plastic that are less than 5 mm in size and can be found in most environments, including the oceans, rivers, and air. These small plastic particles can have negative impacts on wildlife and the environment. In this review of the literature, we analyze the presence of microplastics in various species of wildlife, including fish, birds, and mammals. We describe a variety of analytical techniques, such as microscopy and spectrometry, which identify and quantify the microplastics in the samples. In addition, techniques of sample preparation are discussed. Summary results show that microplastics are present in all the wildlife species studied, with the highest concentrations often found in fish and birds. The literature suggests that microplastics are widely distributed in the environment and have the potential to affect a wide range of species. Further research is required to fully understand the impacts of microplastics on wildlife and the environment.

## 1. Introduction

Microplastics (MPs) are synthetic polymers with major dimension of ≤ 5 mm [1]. Microplastics have been found in various environmental media, including soil, water, and air, and have been shown to have negative impacts on wildlife. The particles occur in a large variety of shapes, sizes, colors, and compositions. In accordance with this premise, the microplastic particles are produced and emitted into the biosphere by many physical processes, the majority of which are still being investigated and discovered. Known sources of microplastics include littering, breakdown of larger plastic items, attrition of textiles [2], microbeads used in personal care products such as face scrubs and body washes, tire and road wear particles [3,4,5], 3D printers [6,7], and even household laundering [8,9]. Microplastic particles are generally considered chemically inert to degradation, though fascinating surface chemistry and adsorption may occur [10,11,12].

Global plastic production has almost doubled compared to two decades ago and most of the plastics are ending up in landfill, incinerated, or leaking into the environment, with only 9% successfully recycled globally. In developed countries, some plastics are recycled, whereas in low-income countries, no advanced equipment for recycling exists. Thus, most of the plastics are burned by the roadside, before washing out in waterways, and contaminating the oceans, ponds, lakes, or rivers. Moreover, in developed countries most of the plastic recycling methods are chemical recycling, incineration, or downcycling, which do not mitigate the microplastic pollution risk—but rather may create more toxic substances from the plastic end-products. Yusuf et al. [13] investigated this and reported that toxic byproducts released from incomplete or faulty incineration of plastic, such as volatile organic compounds (VOCs), polychlorinated biphenyl (PCBs), heavy metals, polycyclic aromatic hydrocarbons (PAHs), and other gaseous emissions in the environment may possibly cause serious health problems to the human body, such as cancer, respiratory diseases, neurological disorders, hemolytic anemia, cardiovascular diseases, and suppression of the immune and nervous systems. Chemical recycling or incineration are not the permanent solution to plastic pollution management, and minimizing unnecessary plastic use, promoting alternative materials, and reducing plastic production are more viable alternatives.

In aquatic systems, microplastic wastes have been proven to absorb and release chemicals that can be harmful to aquatic life [14,15,16,17]. They have also been shown to transfer these chemicals up the food chain to higher trophic levels, potentially affecting the health of species at all levels of the ecosystem [18]. In addition, microplastics have been found to affect the behavior of aquatic species, including altering their feeding habits and reducing their reproductive successes [19,20,21,22]. On land, microplastics have been found in the guts and feces of a variety of wildlife, including birds, small mammals, and insects. The ingestion of microplastics has been shown to have negative impacts on the health of these species, including reducing their body conditions and altering their immune system functions [23,24,25]. The impacts of microplastics on wildlife extend beyond just aquatic and terrestrial environments. Microplastics have also been found in the atmosphere, and the potential impacts of airborne microplastics on wildlife are not yet fully understood [26,27,28].

Despite the growing body of research on the presence and potential impacts of microplastics on wildlife, much remains unknown about their distribution, fate, and transport in the environment. It is however estimated that without an immediate reduction in emission rate, microplastic pollution in the world’s oceans will more than double to 3 Mt a year in 2040 [29]. Given that environmental lifetimes are believed to range from ka to Ga, and given the increasing rate of microplastics’ production, microplastic pollution presents an emerging threat to the one—health landscape.

In this brief review article, our team summarizes approaches for the chemical characterization and analysis of microplastic particles and then outlines select recent literature describing the prevalence and impact of microplastics noted in wildlife, with the goal of improving knowledge of analytical chemistry related to microplastic detection and increasing awareness of the problem. We conclude that more research is needed (and should be supported) on microplastics and their impacts. Research related to the health impacts of MPs—especially in terms of function of size, routes of human and animal exposure, patterns of biodistribution, how MPs degrade in the environment, standardized methods to analyze MPs, and standard reference materials—all represent areas of major gaps in knowledge. While several review articles on microplastics exist, this work is the first to consider methods for chemical analysis, and microplastics’ impact on wildlife—both areas of major knowledge gaps. In addition, the manuscript identifies key areas of gaps in knowledge for future research. In addition to supporting additional research on microplastics, the world’s governing bodies should promote the development and debate of remedial solutions.

## 2. The Chemical and Physical Characterization of Microplastics

The identification of microplastics often involves a multistep procedure. First, possible microplastics are physically separated from samples after digestion, by filtration or gravimetrically through density (floating) [30,31,32]. Next, several microscopies, including optical, fluorescence, and scanning electron microscopy, are used to begin the process of identification. The suspected microplastic is then tested chemically to validate composition [33]. Large numbers of microplastic particles can be screened quickly and effectively with fewer chances of misidentification mistakes using the combination of optical microscopy followed by instrumental techniques providing chemical information. A major theme regarding the chemical and physical characterization of microplastic particles is that no single technique provides sufficient information for comprehensive characterizations of MPs [34]. Thus, several techniques are typically used in conjunction with one another for successful analysis. The following paragraphs outline common sample preparation steps and several common methods of analysis.

### 2.1. Sample Preparation

**Figure 1:**
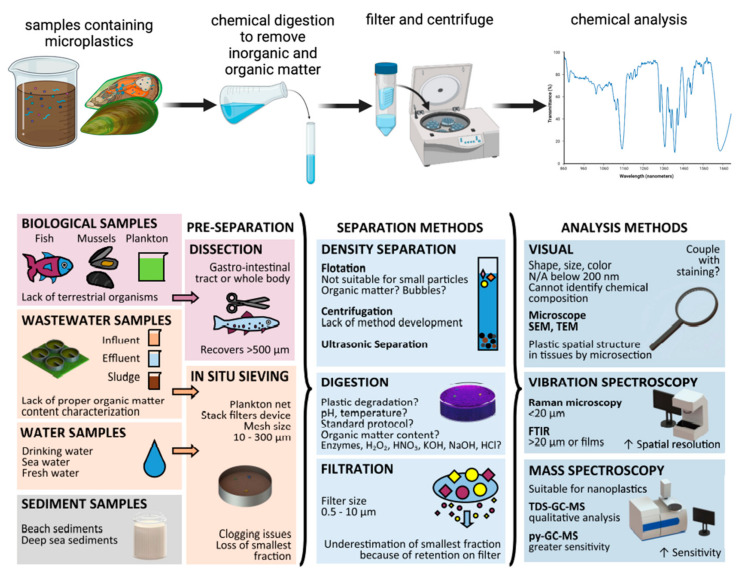
**Top**—General workflow for microplastics analysis. Samples are initially digested to dissolve and remove inorganic and organic materials before gravimetric or filtration-based separation. Finally, chemical analysis of microplastics can be achieved. **Bottom**—Overview of microplastics and nanoplastics separation and analysis methods in simple and complex matrices. Figure reproduced from [35] with permission of the publisher.

The analysis of microplastics present in animal tissues, water samples, or air presents substantial analytical (measurement) challenges since the micron-sized polymeric particles are present at ultratrace mass loadings. The analysis presents the proverbial needle within a haystack problem. In virtually all experiments, sample preparation steps are crucial to the analysis workflow. The general workflow is illustrated in Figure 1 below.

Microplastics are usually collected from the samples by sieving or dissection from animal tissue of interest. Next, most samples will be treated with reagents to chemically digest or dissolve the matrix such as KOH, HNO_3_, or H_2_O_2_ before trying to separate the microplastics by filtration, or gravimetrically by utilizing differences in density [36,37] with or without centrifugation [38]. Fortunately, the chemical stability of the microplastic particles often allows analysts to dissolve and digest various matrices while leaving the polymeric particle intact [39]. However, care must be taken—especially when using nitric acid. One of the common methods used to digest samples is the use of a solution of concentrated (30–35%) hydrogen peroxide to remove organic matter [38]. In one such work by Xiong Xiong et al., digested residues of water samples were mixed with 20 mL of 30% hydrogen peroxide at 60° Celsius for 72 h to digest organic material, then the polymer particles in the digested samples were separated by density overnight using saturated sodium chloride solution (D = 1.2 g/mL) [36]. In another study, microplastics present in air samples were collected and the sample filters were washed before digestion in 35 mL of 30% H_2_O_2_ at room temperature for 10 days to eliminate organic materials. The remaining H_2_O_2_ solution was then vacuum-filtered through filter paper with a 2-micron pore size to remove any remaining particle matter. Next, 50 mL of a saturated ZnCl_2_ solution with a density of 1.6 to 1.8 g/cm^3^ was added to each filter, and the mixture was agitated for five minutes at 350 rpm. Then, the samples were allowed to remain still for one full hour before being centrifuged for 3 min at 4000 rpm to collect all microplastics [38].

Sample preparation workflows for animal tissues are quite similar. For instance, when detecting microplastic in mussels, Jiana Li et al. first collected the mussel samples in conical flasks [40]. Each conical flask then received 200 mL of 30% H_2_O_2_, the bottles were covered (with foil), and the bottles were then oscillated at 65° Celsius and 80 rpm for 24 h, followed by 24–48 h at room temperature, depending on the soft tissue’s state of digestion. Once the digestion fluid seemed clear and no visible particles were present, digestion was stopped [40]. Al-Azzawi et al. have carefully studied digestion environments for microplastic analysis and found that H_2_O_2_ promoted by the Fenton mechanism are superior choices for organic matter removal from microplastic samples while not affecting the tested polymers [41]. One study employing this approach is from Chaudhari & Samnani [42]. In this work, sampled water was filtered to remove the solids. To create the solid mass, the substance was left to dry at room temperature for the whole night to break down organic material within a solid bulk. The sample was then mixed with 20 mL of 30% H_2_O_2_ and 20 mL of an Fe catalyst solution. The sample was then heated at 75° Celsius until the bubbles stopped expanding. To make the aqueous solution denser, 6 g of salt (NaCl) was added (5 M NaCl conc.). Microplastics float on the surface due to differences in density and were separated using a density separator. The filter was used to catch the floating plastic, which was then air-dried, and the plastic was removed. The collected sample was then utilized for spectroscopic identification [42]. Gang Li et al. used an alternate tissue digestion approach [43]. For this work, the tissues were heated to 60° Celsius for 36 h while submerged in 10% KOH. An organic membrane was then used to filter the solution. The membrane’s remaining fishbone fragments were pried off with tweezers. Following that, the membrane and the remaining solids were placed in a glass bottle filled with a saturated NaCl solution to suspend and separate by particle densities for four hours.

In summary, the previous literature suggests that before the chemical analysis of microplastics, samples must be chemically digested, and the most common solution used for this purpose is the 30% H_2_O_2_. Separation by density (floating) for microplastic particles is a practical and common step to easily purify microplastics prior to chemical analysis. In general, the protocol of sampling, filtration, chemical digestion, and density separation is routinely used for the sample preparation steps of microplastics analysis [44].

### 2.2. Optical Microscopy

**Figure 2:**
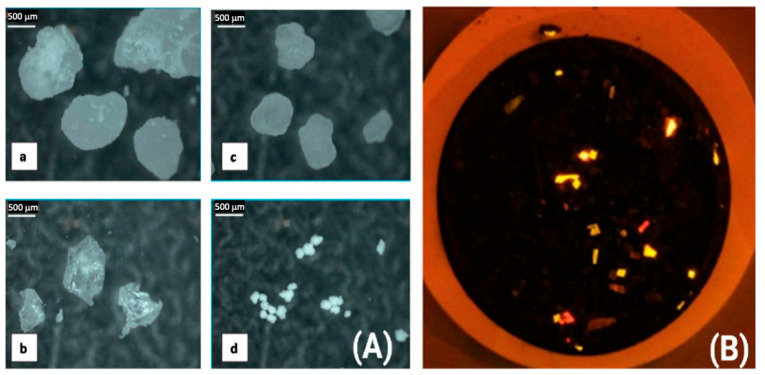
(**A**) Optical microscope images of microplastic particles: (**a**) Polyethylene (PE) particle; (**b**) polyethylene terephthalate (PET) particle; (**c**) polypropylene (PP) particle; (**d**) polyvinyl chloride (PVC) particle. (**B**) Microplastics of six different polymer types, dyed with Nile Red (1000 μg·mL^−1^ for 30 min). Figures reproduced from Abbasi et al. [38] and Maes et al. [46] with permission of publishers.

After separating MPs from the sample of interest, the next step is physical and chemical characterization. Visual inspection is the quickest and most popular way to identify suspected microplastic particles. With the aid of optical microscopes, microplastic particles’ size, shape, and color can be characterized. This method has the advantage of being the simplest, lowest cost, and allowing for the largest diversity of microplastic to be detected in terms of size (primary diameter or length), color, and form (as fiber, film, fragment, and spherule). As an illustration, Figure 2A presents visual features of microplastic particles observed through a Carl-Zeiss binocular microscope [38]. This is the very first stage of plastic screening for all samples. Optical microscopy is utilized as a prescreening method to reduce the number of particles that are required to be examined by SEM or alternate methods. By using optical microscopy, it is facile to identify non-plastic particles, such as organic plant or animal remnants and even some shells, and these were therefore omitted from the follow-up investigation [45].

However, a significant caveat of visual sorting is demonstrated in the work of Frère et al., as results show that visual sorting may cause the concentration of microplastics to be underestimated [47]. These authors suggest that a need exists for a second, follow-up analysis to confirm the polymer nature of the particles. Visual identification of microplastics can indeed be very challenging, since the microscopist has little if any basis to optically discern particle composition.

One advance which aids the microscopist has been borrowed from biology—staining microplastic particles with dyes. Plastic particles can be dyed to improve their ability to be detected because dyes adsorb plastic particles (see Figure 2B). Dyes produce different colors which allow us to visually detect the type, shape, and size of microplastic. In the study of Xu et al., [48] the authors investigated staining microplastics with several dyes which adhere to plastic surfaces and turn the particles colored, or in some cases fluorescent. Multiple dyes used include Oil Red EGN, Eosin B, Rose Bengal, Hostasol Yellow 3G, and NR (Nile Red). Investigators soaked particles in the dyes for different durations between 5 min and 66 h. Nile Red was chosen as the optimal stain since it has the highest levels of adsorption and fluorescence intensity. When exposed to blue light, the dye will fluoresce and simple photography with an orange filter is used to find fluorescence emission. Fluorescent particles can be recognized and counted using image analysis. Particles that were as small as a few micrometers can be detected using magnified images that can be recorded and tiled/arranged to cover the entire filter area. Interestingly, Nile Red’s solvatochromic properties provide the opportunity for plastic categorization based on the surface polarity traits of identified particles. It was established that an incubation period of between 5 min and 66 h and a dye concentration of between 1 and 1000 g mL^−1^ were ideal for visibility. A working concentration of 10 g mL^−1^ produced a nice balance between background signal, visibility, and speed [46].

In the work of Taghizadeh-Rahmat Abadi et al., microplastics present within the digestive tract of commercial Kutum fish sampled from the Caspian Sea were considered [49]. To validate the visual assessment, a subsample of 24 microplastic objects were randomly chosen and stained with Nile Red (NR) after being observed and counted under a stereomicroscope. All samples that were seen were fluorescent. Both blue and green fluorescence made NR-stained microfilaments, fragments, and microbeads visible, with the first one having a more pronounced appearance. All MP with a darker background could be distinguished by their green fluorescence. Bright yellow to red fluorescence (under the blue and green filters, respectively,) was sufficiently produced for MP by staining with NR/methanol solution. The authors were able to establish that all 24 objects observed were MPs using the Nile Red staining procedure.

### 2.3. Electron Microscopy

An additional technique commonly used to characterize the size, shape, and chemical composition of microplastics is Scanning Electron Microscopy (SEM). This technique is used to image and measure objects with different diameters ranging from millimeters to nanometers in size [50]. The SEM technique is capable of nanometer spatial resolution because the deBroglie wavelength of the electron beam used to probe a sample is far less than optical wavelengths, and as such, the optical diffraction limitation is overcome. The electron beams used by scanning electron microscopes (SEMs) can produce images of samples with a resolution of just a few nanometers. Thus, SEM helps observe tiny particles that an optical microscope may miss [51].

**Figure 3:**
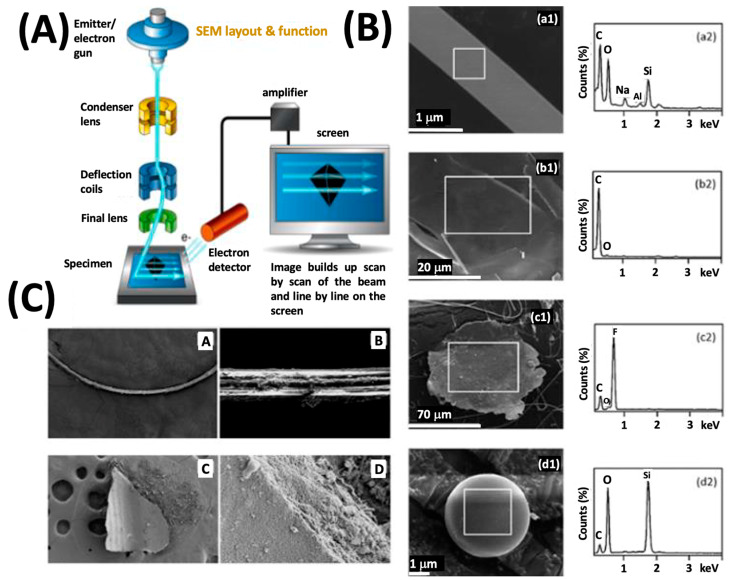
(**A**) Schematic of an SEM instrument [54]. (**B**) SEM images (**a1**–**d1**) and EDX spectra (**a2**–**d2**) from suspect microplastic particles isolated from marine organisms. SEM with EDX allows investigators the ability to exclude particles from being microplastics, as is case of object (**d1**), which appears to be silica [51]. (**C**) SEM images from microplastic (**A**–**D**); SEM images of microplastic debris extracted from the stomach contents of *Bursatella leachii* specimens [55]. Figures have been reproduced with permission of publishers.

A schematic of an SEM is illustrated in Figure 3A. Within the device, the electron source’s filament emits electrons, which are then collimated into a beam. The electron column’s set of lenses then focuses the beam on the sample surface [52]. SEM offers outstanding depth of field, high resolution, and strong sensitivity over a wide range of materials, overcoming many of the traditional limits of light microscopy [53]. The electron optics of an SEM are designed to produce a tiny volume electron probe at the specimen focal plane by demagnifying the tiniest virtual cross-section of the electron beams close to the cathode. A voltage of 0.1 to 50 keV between the cathode and anode accelerates the electrons released from thermionic, Schottky, or field-emission cathodes. Then, the electron beam is swept across the sample while recording the electrons that bounce back (scattered) or are transmitted.

SEM can be combined with a technique known as energy-dispersive X-ray analysis (EDX) in which X-rays are produced during the samples’ irradiation with the electron beam and the signal produced provides chemical information. EDX analysis works because the electron probe beam hits the inner core-shell electrons of an atom, knocking off an electron from the core-shell. This creates an electron ‘hole’ or a vacancy a higher energy electron will rapidly fill. As the electron moves from the outer higher-energy to the inner lower-energy shell of the atom, the energy difference must be released to the surroundings, in the form of an X-ray. The energy of this X-ray is unique to the specific element. Thus, collecting these X-rays and analyzing them allows elemental analysis to be performed upon the sample.

Figure 3B illustrates SEM images of fiber particles and the EDX spectra which provide evidence for a variety of particle compositions for suspect microplastic particles isolated from marine organisms. The presence of an abundance of carbon and oxygen, along with fluorine, may suggest synthetic origins. In the study of Ding et al. [51], the authors used SEM with EDX to screen particles collected from marine organisms for shape and size along with chemical composition. Particles containing an abundance of carbon and/or oxygen may be synthetic polymers, but follow-up analysis using vibrational spectroscopy is required for definitive assignment. EDX spectra reflecting the presence of fluorine such as observed in Figure 3 inset c2 may indicate the presence of synthetic fluoropolymers such as Teflon (PTFE).

The work of Furfaro et al. has achieved similar results when considering the stomachal contents of *Bursatella leachii* as the sample [55]. These authors report that surface texture created by environmental exposure is a key clue that can be used to screen for microplastics by electron microscopy. Degradation, pitting, and abrasion signs on the particles surface suggesting mechanical weathering or abrasion. EDX analyses were used to screen for likely microplastics and rule out nonplastics. Since it is known from the literature that the most common kinds of plastics such as polypropylene (PP) and polyethylene (PE) exhibit a very strong carbon peak in the EDX spectrum. These authors suggest consulting library spectra for typical MP polymers for tentative identification. However, the EDX technique is fundamentally not molecularly specific, so definitive identification by EDX is not technically possible by library searching or matching. Nonetheless, SEM with EDX allows the collection of screening data to rule out certain particles from being MP and the high spatial resolution the technique affords provides information about the shape and surface texture of particles, which may provide a trained analyst’s eye cues as to origin and history.

### 2.4. FTIR Microscopy

Combining optical microscopy and/or SEM with vibrational spectroscopy can reveal the molecular composition of plastic particles. The signal strength obtained, however, depends on the size of the particles being examined, and typically it is crucial to separate the suspect microplastic particle from sample matrix to avoid interfering peaks from appearing in the spectrum [35].

**Figure 4:**
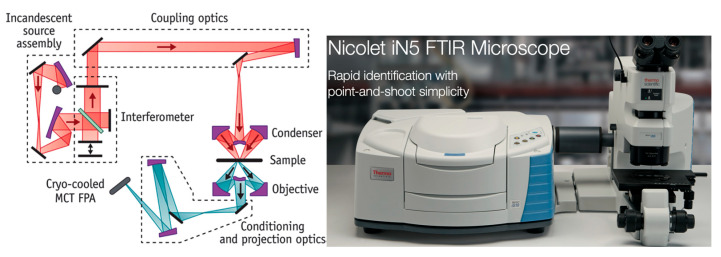
**Left**—Schematic of an FTIR microscope. Reprinted with permission from the March/April 2014 edition of BioOptics World Copyright 2014 by PennWell. **Right**—Photograph of a modern FTIR benchtop microscope. Photograph provided by Thermo Fisher Scientific.

An effective and widely used method for identifying microplastics is FTIR microspectroscopy. The signal is dependent on a change in molecular dipole moment occurring during a molecular vibration. By absorbing IR light, molecules are raised to a higher vibrational state which correlate specifically to types of bonds present in molecules under study. In contrast to SEM-EDX, IR spectroscopy is a very potent technique that offers fingerprint information about the sample’s molecular makeup and specific bonds present [56]. Optimally, an FTIR microscope will be used for rapid spectra acquisition of microplastic particles. However, more conventional equipment can be used as well if a sufficient sample exists and can be prepared for analysis. A schematic of an FTIR microscope and photograph of a commercially available instrument is presented in Figure 4. The spatial resolution of FTIR microscopes is only roughly 10–20 μm, but this is wavelength-specific and constrained by the well-known diffraction limit. Ideally, the sample must be deposited onto an IR-transparent substrate and have a minimum thickness of about 150 nm for FTIR to work well. Due to these drawbacks, FTIR works best for individual particles larger than 20 μm. Agglomerates or films of smaller particles can still be examined [35]. Since it is more effective than other techniques at detecting microplastic particles as small as 20 µm, micro-FTIR spectroscopy (micro-FTIR) is an excellent technology for identifying airborne microplastics [57].

**Figure 5:**
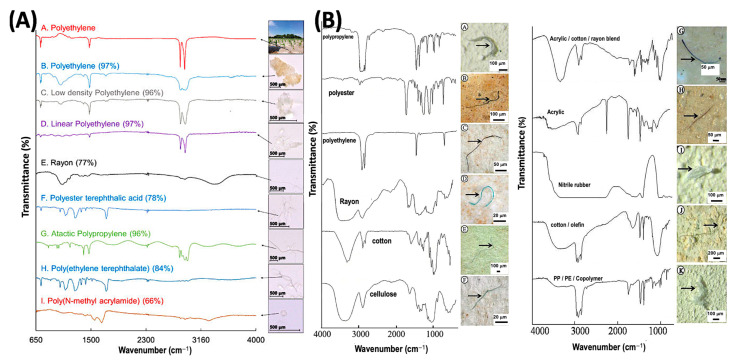
(**A**,**B**)—FTIR spectra and optical images of MPs of various compositions. Characteristic peaks differ based upon MP composition. Figures have been reproduced from Refs. [40,58] with permission of publishers.

Between the visible light and microwave parts of the electromagnetic spectrum is the zone known as infrared radiation (IR). While the IR region extends from roughly 700 nm–1 mm in wavelength, FTIR spectroscopy for molecular fingerprinting is generally applied in a limited range of frequencies between 4000–400 cm^−1^ (wavenumbers) because it is this ‘fingerprint’ region that has significant practical value for identifying bonds within molecules. Different materials’ infrared spectra differ depending on their chemical makeup, so it is possible to see the presence of various bonds or groups of atoms (functional groups) and, consequently, determine composition. To demonstrate, consider Figure 5 below which illustrates optical images and FTIR spectra of a variety of microplastics particles of different compositions. Carefully consider the wavenumbers at which dips in the transmission spectra occur. Polyethylene exhibits reasonably sharp spectral absorbance features near 3000 cm^−1^; however, in contrast, cotton’s spectrum in this region is very broad. An infrared spectrum can be used to differentiate between these two materials and/or confirm identity of a MP. The three FTIR spectral bands most used for microplastics analysis are typically 4000–2750 cm^−1^, 2750–1850 cm^−1^, and 1850–700 cm^−1^. Within the fingerprint region (1850–700 cm^−1^), there are differences in strength and specificity (diversity of signal) which can be exploited to differentiate between various plastics.

One work which demonstrates this is that of Chaudhari and Samnani [42] who used FTIR to detect the presence of microplastics. After comparing the FTIR spectrum results with an electronic library of spectra, the presence of PVC (polyvinyl chloride), PP (polypropylene), PET (polyethylene terephthalate), PS (polystyrene), HDPE (high-density polyethylene) and LDPE (low-density polyethylene) were found present in the sample. Thus, while suspected microplastic particles can be identified visually through optical microscopy, their chemical identity is often verified using FTIR spectroscopy [59].

In the work of S.L. Wright et al. [60], an FTIR microscope was used to count microplastic particles and identify their composition concurrently. This was accomplished by utilizing an FTIR system that includes a mercury cadmium telluride (MCT) detector with liquid nitrogen cooling. The authors were able to identify 15 different polymeric microplastics present in air samples collected via deposition in London. The authors also determined MP deposition rates ranging from 575–1008 microplastic particles/m^2^/day. The MPs observed were of various shapes, but fibers accounted for the vast majority (approx. 92% of the total).

The work of Edo et al. [61] demonstrates use of a more conventional experimental apparatus for FTIR spectroscopy of MPs. Herein, a micro-FTIR with a mercury cadmium telluride (MCT) detector was used to record FTIR spectra in microtransmission mode (not a microscope) because it allowed for highly sensitive observations in the mid-infrared. Samples were visually segregated and placed on KBr pellets, and the following measurement parameters were used for the microtransmission mode: spot 50 μm 20 scans, 8 cm^−1^ resolution, and 4000–550 cm^−1^ spectral range. The apparatus allowed the authors to detect the presence of 12 different anthropogenic polymers or groups of polymers with a predominance of polyethylene, polypropylene, polyester, and acrylic fibers present within the wastewater effluent of Madrid, Spain.

The work of Heshmati et al. also reports use of a conventional attachment for FTIR analysis, the attenuated total reflection (ATR) Fourier transform infrared (ATR-FTIR) approach. After collecting microplastics from fish sampled from the Qarasu River of Iran, the ATR-FTIR sample cell was used to collect IR spectra with an average of 64 scans, at a very typical resolution of 4 cm^−1^ from 450 cm^−1^ to 4000 cm^−1^ [62]. The authors determined that 94% of fish had MPs present, with polystyrene, polyethylene, and nylon being most abundant. Fibers were the most abundant particle type (85.12%), followed by fragment (12.32%), foam (0.77%), film (1.21%), and microbeads (0.56%), respectively.

In another work considering water from the Tamsui River in Taiwan, Wong et al. [63] concluded that microplastics are largely generated from land-based sources since MPs particle concentrations peaked after rain events. FTIR spectra of the samples were collected using the attenuated total reflection Fourier transform infrared (ATR-FTIR) microspectroscopy approach. FTIR spectra of samples were acquired with 128 scans with standard 4 cm^−1^ resolution range of 4000–650 cm^−1^ using a Nicolet 6700 FTIR coupled to a MIRacle™ single reflection germanium ATR cell manufactured by PIKE (Fitchburg, WI, USA).

This sampling of works summarizes the use of FTIR spectroscopy for the chemical characterization of microplastic particles collected from a variety of sources. By no means should this brief overview be construed as being a comprehensive review of FTIR measurements in the field of MPs, but rather an overview of the technique and the information it provides. FTIR remains a very valuable technique for MP identification—particularly when used in microscopy mode. FTIR allows rapid (minutes) determination of the chemical composition of MP particles.

### 2.5. Raman Microscopy

**Figure 6:**
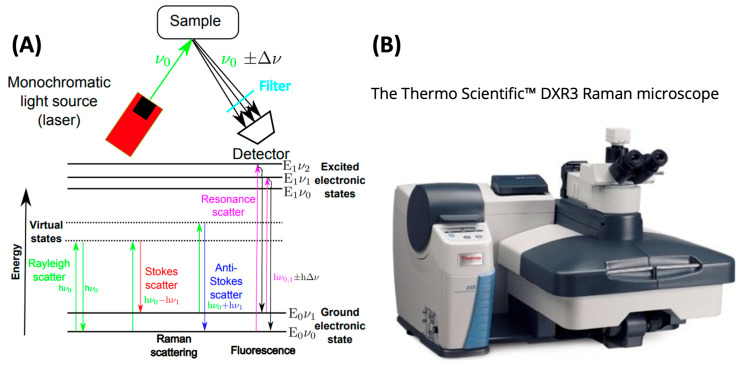
(**A**) Schematic of the fundamental principles of Raman spectroscopy, highlighting the main scattering and fluorescence excitations of a sample after it is excited using a monochromatic light source of frequency equal to ν_0_. The Raman stokes line experiences a frequency shift of Dν that corresponds to the difference between vibrational energy levels. (**B**) A benchtop, commercially available Raman microscope. (**A**) from Ref. [67] via Creative Commons license and (**B**) from Thermo Fisher Scientific.

Another powerful method of analysis for microplastic particles is Raman spectroscopy. Raman spectroscopy is another form of vibrational spectroscopy; however, in contrast to FTIR, Raman microscopy is more appropriate for small microplastics less than 20 μm. Raman spectroscopy is based on the Raman effect, whereby the frequency of a small portion of dispersed or scattered radiation emanating from a sample differs from the frequency of monochromatic incident light. The basic principle is illustrated within Figure 6A below. Raman spectroscopy involves illuminating the sample with a monochromatic laser beam that interacts with the molecules in the sample and produces scattered light. Raman spectra are produced by the inelastic interaction of the sample molecules electrons and incident monochromatic energy. When monochromatic radiation impacts the sample, it interacts with the sample molecules and scatters in all directions [64]. A small fraction of scattered light experiences a shift in wavelength due to the inelastic nature of the process. Incoming excitation light interacts with a sample optically to produce scattered light, which is then reduced in energy by the vibrational modes of the specimen’s chemical bonds. This process is known as Raman scattering [65].

Light is scattered inelastically by Raman-active materials’ molecules due to their chemical vibrations. The fundamental benefit of Raman microscopy is that amorphous carbon can be found when the entire wavelength range is utilized. As a result, microplastics subjected to UV degradation do not significantly affect their Raman spectra. Since visible light is often used as the incident beam, Raman microscopy has a spatial resolution much better than FTIR—about 1 μm. Particle shape and thickness have no bearing on the measurement either. Raman microscopy is potentially a more sensitive method than FTIR for identifying microplastics because of these benefits. Some materials, nevertheless, fluoresce, which masks the vibrational information contained within the inelastic scattering within a bright background. However, this can be reduced through choosing an incident beam of longer wavelength. Dyeing agents as well as microbiological, organic, and inorganic chemicals also may have a significant impact on the Raman signal [35]. The advantages of Raman spectroscopy include nondestructiveness, quick acquisition, reliability, single-point measurements with good spatial resolution, surface maps of large samples, and the use of spectra as sources of qualitative and quantitative data. The disadvantages include the signal’s susceptibility to changes in measurement parameters and the difficulty of interpreting data, particularly when a complex matrix is present [66].

**Figure 7:**
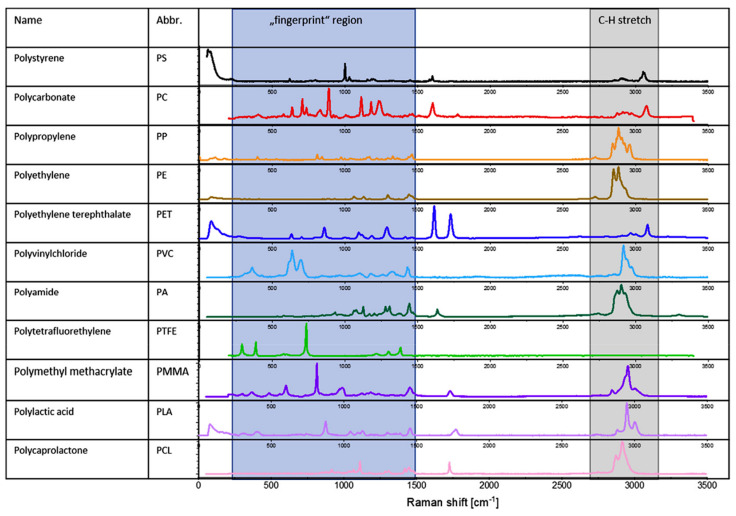
Raman spectra of a variety of synthetic polymers common to microplastics. Figure reproduced from Ref. [68] with permission of publisher.

The micro-Raman technique, which combines collections of Raman spectral information with microscopy, can identify tiny microplastics down to 1 µm, and this resolution cannot be attained by other techniques [57]. Figure 6B depicts a commercially available Raman microscope which can be used for this purpose. Raman spectroscopy provides data which is complementary to FTIR measurements since both use vibrational transitions to provide molecular speciation. In FTIR, the transmittance or absorption of light at a particular wavelength is assessed. In Raman spectroscopy, the data stream is a plot of scattered light intensity vs. the shift in wavelength as measured from the incident beam. The shift in wavelength is the variable which describes the molecular vibrational energy levels. Fortunately, various MP materials exhibit differences in their Raman spectra which can be used to identify polymeric materials. Figure 7 below depicts Stokes-shifted (lower energy) Raman spectra for a variety of materials germane to MP analysis. Clear differences appear in the fingerprint region between 400–1500 cm^−1^ which can be exploited for polymer identification.

Given the rich chemical information provided and excellent spatial resolution, the Raman approach is a popular method for finding and analyzing microplastics in various environments. For instance, in the work of Xiong et al., [36] the authors isolated suspected microplastic particles from various lake waters collected in China. In all, 585 particles were isolated after chemical treatment and detection by counting under an optical microscope. However, only 201 were identified as microplastic using a Raman microscope, accounting for 34% of the total suspect particles. The Raman spectra in the 300–3200 cm^−1^ region were recorded to determine the nature of the compounds. To confirm the polymers present, the Raman spectra of the samples were compared with the library of Raman spectra and the spectra of standard polymers. The Raman spectroscopy identified six types of common microplastic polymers: polyethylene (PE), polypropylene (PP), polyethylene terephthalate (PET), polystyrene (PS), polyamide (PA), and polyvinyl chloride (PVC). The authors determined that 87–750 MP items per m^3^ of water were present in the natural waters tested.

The study of van Cauwenberghe & Janssen [69] considered the abundance of microplastics within marine bivalves typically consumed as food by humans. The authors found 0.3–0.5 particles per gram of tissue for oysters and mussels sampled in Europe. Herein, a micro-Raman spectrometer was used to test a subset of particles recovered to determine the type of plastics present to cover the full diversity of microparticles found. Three spectral windows covering the wavelength range of 80 to 2660 cm^−1^ were used to record high-resolution Raman spectra using a spectrometer operating at a diode laser wavelength of 785 nm. Unfortunately, the authors report difficulty in determining polymer material because of the presence of dyes used which interfered with their signal.

**Figure 8:**
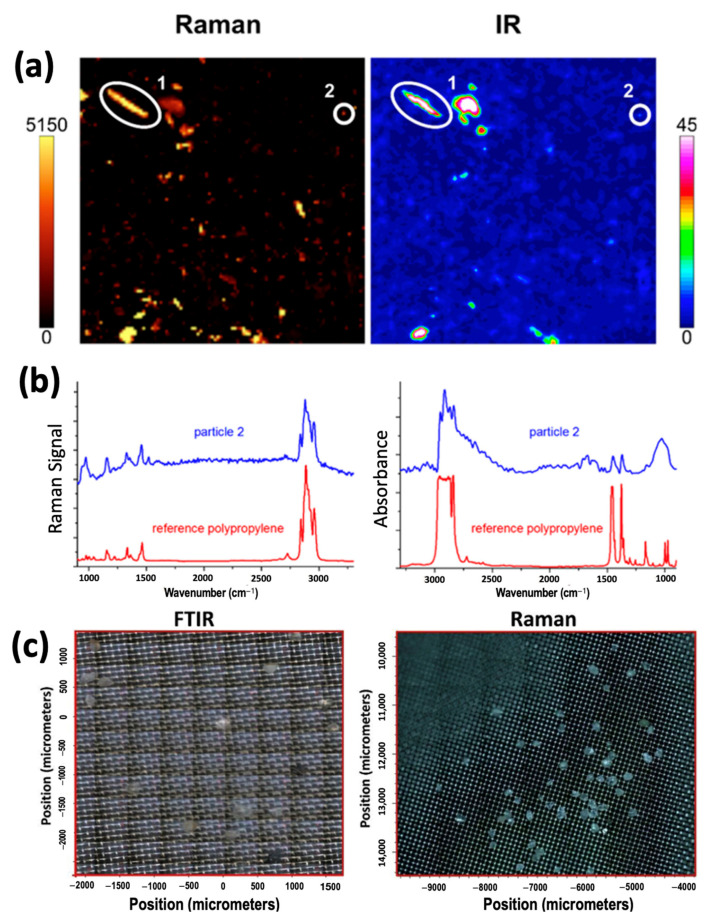
Direct comparisons between Raman and FTIR spectra/imaging of microplastics. (**a**) A Raman image (**left**) and an IR image (**right**) with false coloring denoting the spectral intensity in the 2780–2980 cm^−1^ range. (**b**) A Raman spectrum (**left**) and an IR transmission spectrum (**right**) of particle 2 in comparison with a reference of polypropylene. (**c**) Images of unknown particles extracted from sediment and scanned via both FTIR and Raman microspectroscopy. (**a**,**b**) reproduced from Ref. [70] with permission from Springer Nature; (**c**) reproduced from Ref. [48] with permission of publisher.

Since FTIR and Raman spectroscopy are complementary vibrational spectroscopic methods, their data can be directly compared. Figure 8a,b depict Raman images and FTIR images with false coloring denoting the spectral intensity in the 2780–2980 cm^−1^ range. Figure 8b depicts the corresponding Raman spectrum (left) and IR transmission spectrum (right) of a selected particle in comparison with a reference of polypropylene. Clear similarities exist between the FTIR and Raman spectra, with polymers producing peaks at similar wavenumbers as expected. We also notice the higher spatial resolution afforded by the Raman technique, as the nuances of the shapes of particles imaged are better defined within the Raman image. Figure 8c also depicts the higher spatial resolution available for Raman spectroscopy as compared to FTIR as unknown particles of varying size were extracted from sediment samples and scanned via both FTIR and Raman microspectroscopy. The image mosaic of the FTIR instrument was acquired with an aperture size of 100 μm × 100 μm and a step size of 100 μm × 100 μm, while the image montage of the Raman instrument was collected under 10× microscope objective. As shown in Figure 8c, the FTIR image mosaic presents a lower resolution compared to the Raman montage. FTIR imaging may miss plastic particles that are smaller than the aperture size of the spectrometer, but the technique can detect small microplastics as small as 10–20 µm. In contrast to FTIR, Raman spectroscopy uses a laser beam to focus on a much smaller area and can identify microplastics as small as 1–2 µm.

### 2.6. A Summary of Techniques Available for Microplastic Analysis

It is clear no perfect technique exists for MP analysis which can provide both comprehensive chemical identification and high-resolution imaging capability. Nonetheless, certain tools have been adapted for the workflow, and it is desirable to understand the advantages and limitations of each tool. Table 1 summarizes the main advantages and limitations of common tools used for the analysis of microplastics.

In general, optical microscopy is a great first option to visualize suspected MPs. Then, FTIR or Raman can be used to collect data which verifies molecular composition. We note that SEM with EDX is particularly valuable when suspect MPs are chloro- or fluoropolymers such as PVC or Teflon. Chlorine or fluorine will produce a distinct elemental signature in the EDX spectrum which can be used for identification of fluoropolymers. While no single instrumental tool presently available can enable comprehensive analysis of MPs, the use of conventional methods in conjunction offers the analyst a viable toolbox. Further research is needed to delineate and improve upon methods for sample preparation and digestions, which is a crucial part of the MP analysis workflow.

## 3. Microplastics and Wildlife

### 3.1. Microplastics in Aquatic Animals

The pollution of microplastics (MPs) in the marine environment is an emerging threat to aquatic animals. Ece Kılıç et al. investigated microplastic pollution in commercially important fish species Oncorhynchus mykiss Rainbow trout, *Sparus aurata* Gilthead seabream Linnaeus, and *Dicentrarchus labrax* European seabass from Turkey [71]. These authors found 50–63% of fish tested had MPs within their gastrointestinal tracts. Among all species of fish tested, rainbow trout contained the highest amount of MP (1.2 MPs particles per fish), followed by European seabass (0.95 MPs per fish) and Gilthead seabream (0.8 MPs per fish). About 80% of the MPs detected were fibrous in shape and were made of polyethylene (25%), polyester (20%), and polyamide (10%). Most microplastic particles observed were black (61%) or blue (27%) in color.

The improper disposal of surgical masks can cause substantial MP pollution and the potential for severe adverse effects on the marine Copepod (*Tigriopus japonicus*). Jiaji Sun et al. investigated Copepod fed by polypropylene common to surgical masks (PP-SMs) [72]. Polypropylene is a common material used globally during the COVID-19 pandemic to produce surgical masks. A significant decrease of fecundity in Copepod was observed in the treatment group of 100 MPs/mL, with an average of 19.96 ± 5.86 offspring in the first four broods, which could be because of the inhibition of embryonic development and reduction of food ingestion by microplastics. In contrast, the fecundity in control, and treatment groups of 1 and 10 MPs/mL was 25.57 ± 5.04, 26.50 ± 4.83, and 21.79 ± 5.36 offspring, respectively. Results suggest MPs may adversely impact the Copepod’s population, threatening their ecosystem’s balance.

Microplastic pollution has increased substantially in the past few years and now represents a major global environmental issue. Wootton et al. investigated wild-caught fish purchased from different seafood markets in different regions of Australia which are used for human consumption [73]. After the assessment, they found an average of 35.5% of fish samples had at least one piece of microplastic in their gastrointestinal tract. South Australia had the highest percentage of fish with plastic (49%) and Tasmania had the lowest (20%). The average microplastic load was 0.94 pieces per fish but ranged from 0 to 17 pieces, with polyolefin identified as the dominant polymer group.

**Figure 9:**
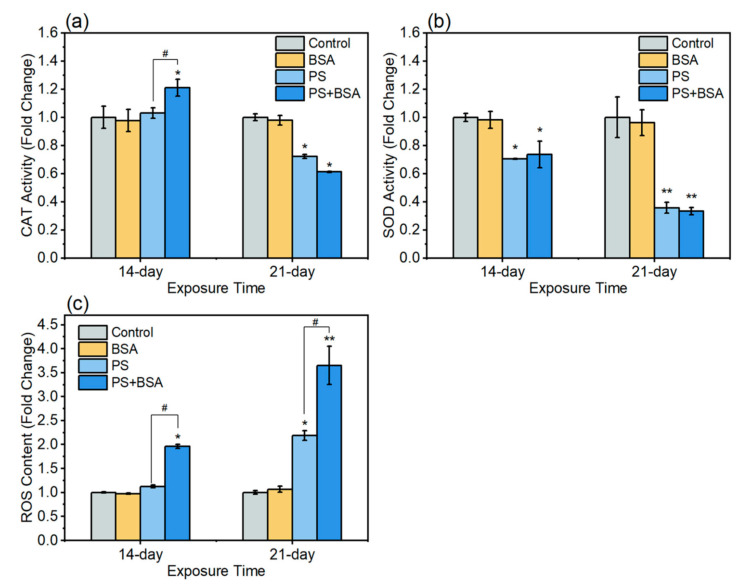
CAT activity (**a**), SOD activity (**b**), and ROS content (**c**) in zebrafish intestines after 14- and 21-day exposures to BSA, PS, or PS + BSA. * *p* < 0.05 and ** *p* < 0.01 indicate significant differences between the treatment groups and the control group. # *p* < 0.05 indicates significant differences between the two groups indicated. Figure reproduced from Luo et al. [74] with permission.

The hazardous effect of microplastics interacting with biomolecules is sometimes overlooked. Microplastics interact with biomolecules such as apocrine protein resulting in the development of protein-coated microplastic complexes in the zebrafish body. Such biomolecule-coated MPs may affect normal physiology or allow MPs to escape natural removal mechanisms. In 2022, Luo et al. worked on adult zebrafish and demonstrated the harmful effects of polystyrene microplastics (PS), and bovine serum albumin (BSA) coated PS corona complex (PS + BSA) led to prolonged duration of retention in fish guts, impairing food consumption and nourishment [74]. The total food intake ratios in zebrafish after exposure to BSA or PS were reduced by 0–21.7% and 7.1–23.0%, respectively, when compared to the control group at the five observation timepoints (1–5 min). Total food intake ratios in zebrafish were reduced by 64.3–69.6% after exposure to PS + BSA at the five observation timepoints (1–5 min), indicating that total food intake was approximately only 30% of that of controls at the 5 min timepoint for the corona complex. As a result, when zebrafish were exposed to PS + BSA, their food intake was significantly reduced compared to the control, BSA, and PS groups. PS + BSA exposure contributed to lower Keap1-Nrf2-ARE antioxidant signaling pathway transcript and protein levels as opposed to PS exposure alone. Figure 9 reports that exposure to PS + BSA deteriorated the potent antioxidant action of related enzymes and led to the formation of reactive oxygen species (ROS), potentially causing significantly more gastrointestinal damage than PS exposure alone. Consider Figure 9a which demonstrates a significant change in the level of catalase after exposure to MPs. Catalase is crucial for the bioregulation of hydrogen peroxide in organisms, and alteration of the enzyme’s activity suggests MPs may play a role in altering levels of reactive oxygen species (ROS). Figure 9b is even more concerning, as it illustrates a significant decline in superoxide dismutase (SOD) activity after exposure to MPs. Superoxide (O_2_^−^) is highly reactive and must be regulated in organisms through the action of SOD or it will cause massive cellular damage. The large decline in SOD activity observed by the investigators is of significant concern as it implies that MPs may cause significant cellular level disruption via oxidative chemistry. The authors also found levels of reactive oxygen species (ROS) more than doubled when MPs were administered—confirming suspicions that MPs affect cellular-level oxidative balance. The results presented in Figure 9 are quite troubling since they suggest MPs cause seismic shifts in cellular chemistry within the zebrafish model. Such changes would be expected to exert substantial impacts on the lifeform.

Microplastics (MPs) may make it easier for organic contaminants like hydrocarbons present in oil to reach organisms by adsorbing onto MP surfaces. Gonzalez-Soto et al. investigated mussels that were exposed for 21 days to 4.5 μm polystyrene microplastics alone or with sorbed water accommodated fraction (WAF) of crude oil in two dilutions (25% termed MP25, and 100% termed MP100) [75]. Microplastic exposure was found to alter glutathione transferase and isocitrate dehydrogenase enzymatic activity in the mussels’ body, caused genotoxicity, increased oocyte atresia and basophilic cell volume in digestive tubules, and decreased absorption efficiency. Aquatic animals like mussels are exposed to mixtures of microplastics and polycyclic aromatic hydrocarbons (PAHs) particles that enhance the bioaccumulation and biotransformation of MPs which can enter the food chain of humans and animals that can lead to health hazards to the top predators. PAHs concentrations in control mussels and mussels exposed to MPs alone were close to detection limits and were comparable to PAHs concentrations measured in mussels exposed to MP25 and MP100. The total amount of PAHs measured ranged from 142 ± 39.27 to 188 ± 40.29 ng/g dry weight (control group and MP100, respectively) on day 7 and from 156 ± 11.42 to 247 ± 59.39 ng/g dry weight (MP and control group, respectively) on day 21. Naphthalene was the most common PAH in all cases. Mussels exposed to WAF bioaccumulated many PAHs, particularly on day 7. Total PAH values were higher on day 7 (ΣPAHs2306 ± 372.42 ng/g dry weight)) than on day 21 (ΣPAHs 1229 ± 165.30 ng/g dry weight) which had an increased prevalence of oocyte atresia.

Microplastics also cause intestinal toxicity in *Amphioctopus fangsiao.* In this study, Zheng et al. demonstrated *A. fangsiao* exposed to microplastics at concentration of 100 (low concentration polystyrene microplastics, PS-L) and 1000 (high concentration polystyrene microplastics, PS-H) μg/L for 21 days (about 3 weeks) exhibited physiological changes [76]. These authors found the mean values of reactive oxidative species (ROS) content in the PS-L and PS-H groups have significantly increased (4.19 ± 0.069 and 4.31 ± 0.050 fluorescence intensity/mg protein, *p* < 0.001) in comparison to the control group (3.02 ± 0.013 fluorescence intensity/mg protein). In the case of malondialdehyde (MDA) levels, which is commonly known as a marker of oxidative stress, the authors found 2.53 ± 0.092 and 2.20 ± 0.165 μM/mg protein in PS-L and PS-H groups, but in the control group, it observed 2.59 ± 0.066 μM/mg protein which the authors interpret as the level of lipid peroxidation increasing significantly in high concentration of MPs treatment (*p* < 0.05). Likewise, superoxide dismutase (SOD) (11.80 ± 0.550 and 13.75 ± 0.51 U/mg protein) and catalase (CAT) (11.03 ± 0.650 and 13.12 ± 0.61 M/min/mg protein) activities in the intestines of PS-L- and PS-H-treated octopuses were significantly higher than in the control group (9.34 ± 0.087 mol/mg protein) (*p* < 0.001). Thus, the oxidative stress parameter levels SOD and ROS were significantly increased under micro-PS exposure stress, leading to the imbalance of homeostasis. Due to the oxidative damage caused by micro-PS stress, the antioxidant enzyme activities of SOD and CAT were stimulated and increased the risk of various physiological damages in the octopus.

Microplastics mixed with certain organic contaminants can cause severe effects on aquatic wildlife [77]. Huang et al. investigated the effects of polystyrene combined with organophosphate pollutant chlorpyrifos in zebrafish. They administered fish feed in combination with polystyrene microplastics with the concentration of 50 and 500 μg/g dry weight which was denoted as PSL (low) and PSH (high), respectively, and chlorpyriphos with the concentration of 0.02, 0.2, 2, 20, and 200 μg/g dry weight feed and denoted as CPF1, CPF2, …… CPF5, respectively. Fish were fed twice daily in a 10 L tank with the contaminated feeds at a level of 2% body weight. The accumulation of microplastics in the gut and liver of fish was observed by fluorescent microscope and found MPs were increased over time and reached steady-state after 4 and 8 days. For PSH and PSL treatments, the maximum amounts of MPs were found as 1.5 × 10^4^ items/fish for PSH treatment and 6 × 10^3^ items/fish for PSL treatment. In the liver, they found about 500 and 300 items/individual in PSH and PSL treatments, respectively. Adsorption of chlorpyrifos by MPs followed a pseudo-first-order model, which increased rapidly at first and reached equilibrium at 4 and 8 h for 20 and 200 g/L CPF, respectively. When compared to the control, both single MPs and mixture treatments significantly increased the number of goblet cells (*p* < 0.05). There was a dose-dependent increasing trend of goblet cells with CPF treatments, but only CPF5 (200 g/g) caused a significant change (*p* < 0.05). Microplastic alone had limited effects on SOD activity in both fish gut and liver in most of the cases except that PSH inhibited SOD activity by almost 45% after 14 days of exposure. Contrary to SOD, single MPs significantly affected CAT activity in both gut and liver tissues, but in different ways. CAT activity in the gut increased initially but then decreased with increasing exposure time, reaching a peak at 4 and 7 days for PSH and PSL treatments, respectively. SOD activity in both gut and liver tissues decreased as exposure time increased, with the maximum inhibition reaching 75% (14th day) for gut SOD and 30% (7th day) for liver SOD.

Microplastics in association with zoonotic parasites are a great threat to shellfish health, which in turn, have a risk to enter the human and wildlife food chain by the consumption of affected shellfish. The author Zhang et al. [78] investigated zoonotic protozoan parasites such as *Toxoplasma gondii*, *Cryptosporidium parvum*, and *Giardia enterica* with polyethylene microbeads and polyester microfibers and found that microplastics can increase the bioavailability of parasite and cause severe impacts on shellfish in the marine environment. In this study, the author used 27 bottles (15 treatment bottles, 9 control bottles which are plastic-free, and 3 negative controls) using 30 mL of seawater in each and microbeads and microfibers were tested separately in those bottles. The treatment and control bottles include microfiber treatment bottles (microfibers + parasites), microbead treatment bottles (microbeads + parasites), and negative controls either microbeads or microfibers only). Microplastic treatment bottles (n = 15, 5 per time point) contained microplastics as well as a mixture of 1000 oocysts of *T. gondii*, *G. enterica*, and *C. parvum*. In this experiment, microbead experiment bottles and microfibers experiment bottles contained 0.053 g (940 particles) and 0.000156 g (481 particles), respectively, and they used a concentration of 31 microbeads/mL and 16 microfibers/ mL in the first experiment and a concentration of 31 microplastics/mL in the second experiment. All three protozoan parasites were found on the surfaces of microplastics, including microbeads and microfibers. In the case of microbeads, parasite counts on these plastics increased over time. In contrast, G. *enterica* and *T. gondii* parasite counts in seawater decreased over a 7-day period, but not *C. parvum*. Counts of *G. enterica* associated with microbeads increased significantly (*p* < 0.05) over each testing day. On day 7, *T. gondii* numbers on the microbeads were significantly greater than on day 1, whereas *T. gondii* concentrations in the seawater were significantly lower than on day 1. Results suggest parasites coagulate with MPs in solution, but further work is needed to assess the impact on animal health.

Microplastics are present in the tissues of various wild coastal animals and are of great concern for transmission from infected tissues to the food chain of tertiary animals or humans. The authors Haave et al. [79] investigated samples of the stomach, intestinal wall, liver, and muscle of flounders, cod, seabirds, otters, and seals, and found the highest level of microplastic in the cod liver which may be transferred throughout the food web of wild animals and birds. Local fishermen donated fresh or frozen birds and mammals to the study. The animals were mostly caught as bycatch in fishing nets and crab traps. Three otters (*Lutra lutra*), two sawbill ducks (red breasted merganser; *Mergus serrator*), and one common guillemot (common murre; *Uria aalge*) were delivered to the Norwegian Veterinary Institute (NVI) in Bergen for investigation of both microplastic and health parameters. One harbor seal (*Phoca vitulina*) was autopsied in the open air. Three cod (*Gadus morhua*) and three flounders (*Limanda limanda*) were caught exclusively for the project by Norwegian Hunter and Anglers Association volunteers and delivered fresh to NVI. Three additional otters were sampled for health parameters but were not included in the chemical MP investigation. All animals were autopsied in accordance with NVI guidelines (http://kvalitet/eknet/docs/pub/dok01304.htm accessed on 15 December 2022). Most organs’ tissue samples, including tissues with obvious lesions, were fixed in 10% neutral buffered formalin, embedded in paraffin, and regularly processed into slices of 3–4 mm thickness for histopathological analysis. During the autopsy, evident microplastics and intestinal blockage were checked in the stomach and intestinal contents. A chemical study was carried out to look for the presence of MP in internal organ tissue. All the animals’ livers, stomach walls, intestines, and a large section of muscle were sampled for chemical tests. The largest portion of the pectoral muscle (*M. pectoralis* major) was removed from birds, the central dorsal fillet behind the dorsal fin from fish, and the internal muscle of the back (*M. iliopsoas*) from mammals. Additionally, samples of fish gills, otters’ spleens, and the kidneys of birds and mammals were taken. A lung fragment from the harbor seal was also examined. The animals’ stomachs were opened, and the intestines were cleaned out and examined for large plastics that might have an impact on the animals’ health and well-being. Using a tiny glass funnel, the intestines were rinsed thoroughly with 50–100 mL of saline. Prior to the examination, samples were weighed, wrapped in aluminum foil, and kept frozen at 20 °C. The two sawbill ducks, which are seabirds, had intestines with MPs of PVC and PS of roughly the same concentrations, whereas the guillemot had detectable amounts of MP in the stomach (PVC) and liver (PET), but no MP in the intestinal tissue. However, cod was the only species for which repeated samples were examined. Of all the animals, cods exhibited the highest frequency and tissue concentrations of MP. MP was present in all three tissues—liver, muscle, and intestines—in two of the three cods. Only one sample of muscle and no other tissues from flounders had MP. At least one tissue sample from 8 of the 13 examined animals contained measurable MP levels. Four separate animals had measurable MP levels in their muscles and/or liver, whereas seven of the eight animals had MP in their intestines or stomach walls. The Cod liver has the greatest concentration of MP in any tissue, at 3.4 per gram wet weight.

Polyvinyl chloride (PVC)-type microplastic can alter the antioxidant activities and inhibit the growth rate of fish larvae (*Cyprinus carpio*). Xia et al., 2020, investigated the exposure of PVC in freshwater fish larvae and found microplastics significantly inhibit the growth and inverse relationship found in SOD (superoxide dismutase) and CAT (catalase) activities [80]. A control group (no PVC microplastics) and three further groups (each with varying PVC microplastic contents of 10%, 20%, and 30% microplastics by weight) were set up for the 30-day and 60-day food exposure studies. According to these concentrations, there were 45.55 g, 91.1 g, and 136.65 g of microplastics per liter of water, respectively. Enzymatic activities and protein concentrations were assessed after the 30- and 60-day trials using the livers, intestines, and gills that had been removed from 12 fish in the control group and for each concentration. Four different fish tissues were taken out and mixed. Using an OxiSelect SOD Activity Assay kit from Cell Biolabs (STA-340), a CAT Assay kit from Solarbio (BC0200), a Micro GPX Assay kit from Solarbio (BC1195), and an MDA Assay kit from Solarbio (BC0025) the activities of SOD (superoxide dismutase), CAT (catalase), GPx (glutathione per-oxidase), and MDA (malondialdehyde) levels, respectively, were determined for the livers, intestines, and gills. After 30 and 60 days of exposure, the liver, gut, and gills were examined for SOD, CAT, GPx, and MDA levels. The findings showed that after 30 days of exposure, all treated groups’ liver SOD activity considerably decreased in comparison to the control group. However, compared to the control group, the SOD activities in the gills and intestines were initially significantly increased in the 10% PVC concentration group and thereafter decreased in the other PVC concentration groups. In contrast to the control group, the SOD activity in the livers, intestines, and gills were considerably reduced after 60 days of exposure in all exposure groups. Following a 30-day exposure period, the CAT activities in the intestines and gills were considerably and dose-dependently reduced in all three PVC concentration groups. The 10% PVC concentration groups significantly increased CAT activities in the livers and intestines, which then gradually decreased in the 20% and 30% PVC concentration groups. There were no statistically significant differences in GPx activity in the liver between the control and treated fish (at all concentrations) after 30 days of PVC exposure. After 30 and 60 days of exposure, MDA levels in the livers, intestines, and gills were significantly lower in all treated groups compared to the control group.

### 3.2. Microplastics in Birds

Microplastics are nowadays very extensive pollutants prevailing in seas that enter the plankton forages and zooplankton. The authors investigated 30 storm petrels (*Hydrobates pelagicus melitensis*) in the Mediterranean Sea which foraged with zooplankton, and they found 45% of the sampled storm petrels had previously ingested microplastics via food. They suggested that storm petrels could be used as an effective indicator for microplastic pollution/ingestion in the pelagic seabirds.

Microplastic pollution in terrestrial ecosystem suggests that MPs transform from source to top predators by entering the food chain and soil is the great reservoir of MPs. Nessi et al., 2022 investigated the barn owl (*Tyto alba*) diet which is characterized by predation on synanthropic rodents. Pellets collected from owls contained MPs and were analyzed by micro–Fourier Transform Infrared Spectroscopy (μ-FTIR). Authors found 33% of pellets were containing MPs, mainly macrofibers (88.2%) [81].

**Figure 10:**
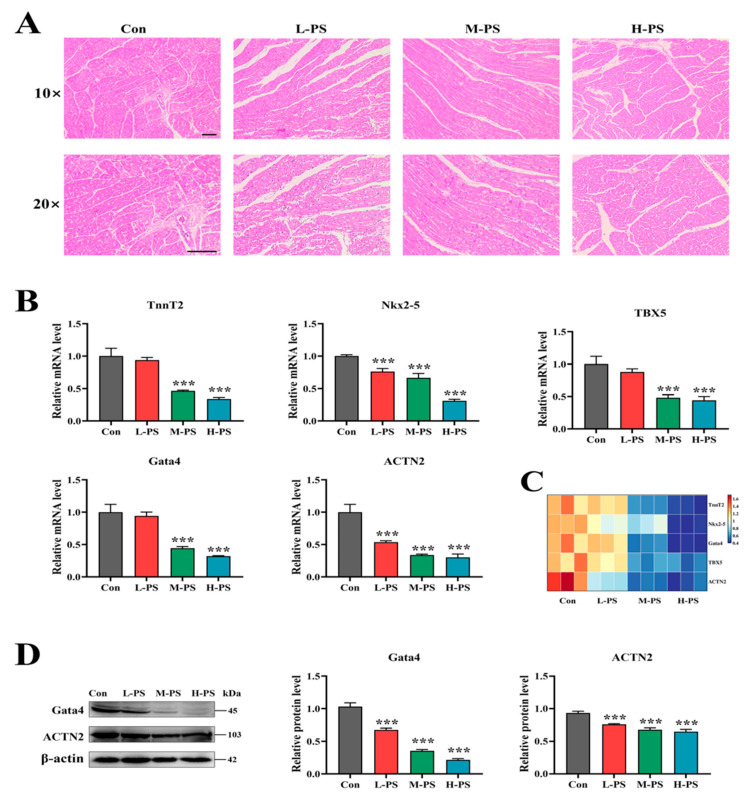
Effects of PS-MPs exposure on myocardial development. (**A**) Histopathological damage to the myocardium at different concentrations of PS-MPs (objective magnification of 10× and 20×). (**B**) Relative mRNA expression level of TnnT2, Nkx2-5, Gata4, TBX5 and ACTN2. (**C**) Heat maps of relative mRNA expression. (**D**) Protein level of Gata4 and ACTN2. The results are presented as mean ± standard deviation (SD), compared to the control group, *** *p* < 0.001. Figure reproduced from Ref. [82] with permission of publisher.

Microplastic pollution can cause myocardial dysplasia by the influence of endoplasmic reticulum (ER) stress-controlled autophagic pathways in terrestrial birds. Zhang et al. [82] developed an in vivo chick model for administering polystyrene microplastics (PS-MPs) in different concentrations (1 mg/10 mg/100 mg/1000 mg per liter of water) to chosen 12-day-old chicken embryos in vitro and then extracting primary cardiomyocytes for investigating the effect of PS-MPs on myocardial development. After a histopathological study, they found loose and irregular myocardial tissue arrangement with large cell gaps and damaged myocardial fiber bundles after PS-MP exposure, as observed in Figure 10. In addition, ER stress markers GRP78, PERK, eIF2α, IRE1, ATF4, ATF6, and CHOP were overexpressed, autophagy-related genes LC3, ATG5, Beclin1, and P62 were down-expressed, and TnnT2, Nkx2-5, Gata4, TBX5, and ACTN2 were downregulated after PS-MPs exposure. The TnnT2 gene expresses cardiac troponin T, a protein involved in the regulation of muscle contraction. Nkx2-5, TBX5, and Gata 4 have been shown to be crucial for the development of the heart in experimental animal models. ACTN2 is responsible for crosslinking filamentous actin molecules in tissues. In addition to the visually observed histopathological damage to the myocardium (Figure 10A), the highly significant, dose-dependent downregulation of the mRNA for these genes reported in Figure 10 suggests MPS administered to the chick embryos had substantial deleterious effects on the heart of the animals tested. So, the authors hypothesized that PS-MPs-induced myocardial dysplasia in birds is driven by the ER-stress-regulated autophagic pathway.

Naturally aged polystyrene microplastics affect the different biomarkers of the small-sized terrestrial bird (*Coturnix Coturnix japonica*). de Souza et al. [83] investigated birds that were assigned in two groups and fed with either 11 and 22 microplastic particles/day/bird, once a day for 9 days, and observed the effects in different organs. The groups were denoted by MP-I for 11 particles and MP-II for 22 particles of MP. The animals in the control group did not receive any type of plastic particles. Results found that significant reduction in body biomass in the MP ingested group and total reactive oxygen species (ROS) levels in muscle and liver in quails are higher in MP-I (average increase of 17.4%) and MP-II (average increase of 23.2%) groups. Also, birds having high MPs showed higher production of malondialdehyde in the liver, intestine, gizzard, and brain, and decreasing effects on hepatic nitric oxide production were found. The antioxidant response marker of the birds exposed to MPs and superoxide dismutase activity in the liver and intestine were decreased by 22.5% and 20.1%, respectively, in relation to the control group.

Macro- and microplastic can cause severe multiorgan effects on seabirds. The paper by Rivers-Auty et al. [84] investigated the impact of ingested plastics on the free-living flesh-footed shearwaters (*Ardenna carneipes*) and after an unsuccessful fledging attempt, the birds were euthanized under permit after being nest-bound and provisioned by both parents for the previous 90 days. Plastic ingestion is common in flesh-footed shearwaters, and severely affected birds are unable to regurgitate plastics before departing for the sea. The birds used in this study were chosen at random along a gradient of plastic exposure, ensuring that there was a control group of birds (n = 8). They weighed and measured birds with a spring scale, a stopped ruler (±1 mm), and exposed culmen (±0.1 mm) and head + bill (±0.1 mm) using Vernier calipers. Plastic items larger than 1 mm recovered from the gizzard and proventriculus were washed, dried, and weighed to the nearest 0.0001 g using an electronic balance. In free-living seabirds, they discovered pathologically significant tissue- and cellular-level sublethal effects of ingested plastics. In comparison to birds that had little to no ingested microplastics, those that had ingested plastics had a higher inflammatory response, more deterioration of the stomach lining, higher tissue damage scores across multiple organs, and a higher density of embedded micro- and nano-plastics in the proventriculus, spleen, and kidneys. These findings demonstrate the negative and insidious effects of plastic pollution on wildlife that have previously gone unnoticed. They found significant effects of inflammation in inferior regions of the proventriculus of the birds and oedema, erythema (redness), and the loss of tissue structure on the epithelial surface of the proventriculus. Water content was used to assess oedema, and the presence of plastic was associated with significantly higher water content in both the superior and inferior regions of the proventriculus.

**Figure 11:**
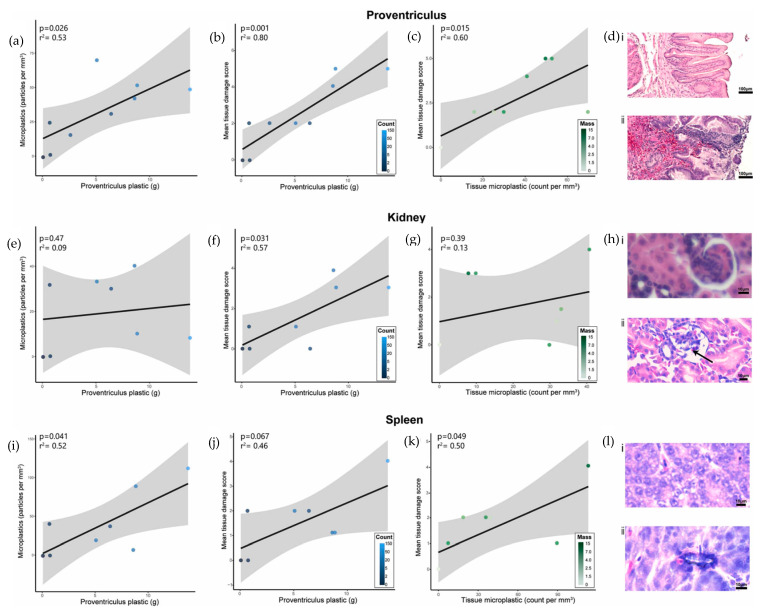
The mass of proventriculus microplastics (>1 mm) correlates with organ microplastics (<1 mm) and organ pathology. Inserts (**a**,**e**,**i**): The correlation between the mass of proventriculus microplastics (grams) with tissue microplastics identified through histology in the proventriculus (**a**), kidney (**e**), and spleen (**i**). Inserts (**b**,**f**,**j**): The correlation between the mass of proventriculus microplastics with mean tissue pathology score (0–5) in the proventriculus (**b**), kidney (**f**) and spleen (**j**). Inserts (**c**,**g**,**k**): The correlation between the tissue microplastics with mean tissue pathology score (0–5) in the proventriculus (**c**), kidney (**g**), and spleen (**k**).Inserts (**d**,**h**,**l**): Histological examples of healthy tissue (i) and tissue pathology (ii) in the proventriculus ((**d**), inflammation and hemorrhage; scalebar 100 μm), kidney ((**h**), collapsed glomerus around microplastic (arrow), scalebar (μm), and spleen. Figure reproduced from Ref. [84] under the Creative Commons Attribution License.

Ingestion of plastic also resulted in a significant loss of rugae, which plays an important role in digestion and nutrient absorption by increasing surface area and allowing the proventriculus to expand. The density of microplastics (particles/mm^3^) in seabird tissues was significantly correlated with the mass of visible plastics recorded in the proventriculus, implying that the mass of visible plastics recorded in the proventriculus can act as a proxy for the number of microplastics detected in the tissues. This finding suggests that the visual method of identifying microplastics in histological sections is accurate for approximating microplastic exposure, as significant levels of particle misidentification would prevent the discovery of the correlation with microplastic exposure. A blinded observer scored the tissue for pathology on a scale of 0–5, with the pathology score being significantly correlated with proventriculus plastic mass as observed in Figure 11 below.

An additional paper has reported the first identification of microplastics in Indonesian seabirds, little black cormorants (*Phalacrocorax sulcirostris*) [85]. The MPs were identified as present within the digestive tract likely due to ingestion of food which is taken mostly from seawater. The author identified 16 microplastic particles of a different type, size, and color and gave an estimation of 320 particles/bird. MPs were mostly films (75%), followed by fiber (18.75%), and fragments (6.3%) in type. Sizes of the microplastic were mostly (68.7%) between 100–1000 micrometer major dimension. The color of the microplastics found was transparent (56.2%) followed by red (18.7%), black (12.5), yellow, and blue (6.2% each). Microplastic color is important due to many animals discriminating against their food choices by color.

Microplastic pollution is truly a growing global crisis and even remote areas like the Arctic are also polluted by microplastics. A recent and first investigation into plastic ingestion by glaucous gulls (*Larus hyperboreas*) in the Arctic region reveals information about the health status of those species in the arctic region [86]. The authors investigated and found microplastics occurred in 14.3% of the bird’s intestines. MPs sampled were mostly polypropylene and polystyrene. This prevalence is less than what was observed in other environments; however, the migration of microplastics to sparsely populated environments is remarkable.

### 3.3. Microplastics in Land Animals

Blackbirds and song thrushes are commonly used as indicator organisms of microplastic pollution in terrestrial environments. One work investigated differently sized microplastics in the gastrointestinal tracts of dead birds which were mostly consisting of fibers (84%) in northeastern Poland [87]. This work found that all the analyzed birds contained microplastic in their gastrointestinal tracts. A total of 1073 microplastic objects were noted, with fibers consisting of 84% and films below 1 mm in size making up approximately 10% of objects. The dominant colors of microplastics were transparent (75%) and brown (14%). The average MP concentration was higher in song thrushes (40.1 objects per bird) compared to common blackbirds (21.9 objects per bird); however, the difference between species was not deemed statistically significant. No seasonal differences or age-related differences in microplastic ingestion in either species were observed. Their findings suggest that thrushes could be used as indicators of microplastic pollution in terrestrial ecosystems. As a result, they recommend the use of such a sampling technique in future work of MP pollutants of wildlife and the ecosystems in which they live.

Microplastics can affect the behavior and physiological condition of animals including chickens. Aoyun Li et al. [88] investigated different adverse conditions in chicken health after administering microplastics. In the study they found microplastic exposure decreased growth and antioxidant ability, impaired chickens’ viscera, and found a significant decrease in alpha diversity, accompanied by taxonomic composition alterations in gut microbiota in chicken that leads to hampering the nutrition metabolism and gut microbial homeostasis in chicken. This study may serve as inspiration for ecologic agencies around the world to regulate the use and disposal of plastic products to reduce environmental pollution. They also discovered a production connection or interaction between the gut microbiota and metabolites, expanding our understanding of the functions of gut-residing bacteria and their metabolites. Furthermore, it demonstrated that the interaction between gut microbiota and metabolites may be one of the mechanisms by which MPs exert their negative effects.

Airway contamination of microplastics can cause severe health problems in mice. The Zha et al. [89] showed that respiratory microbial dysbiosis is induced by microplastics/nanoplastics in mice. There were multiple bacteria (nasal staphylococcus, lung Roseburia, lung *Eggerthella*, and lung *Corynebacterium*) which were potential biomarkers of micro/nanoplastic inhalation for respiratory illness. To some extent, there was association of SAR11_Clade_Ia and SAR11_Clade_II in both nasal and lung microbiota in microplastic contamination that aggravates the condition and they found that microplastic had a stronger influence on the lung microbiota than nanoplastic. However, this report represents a single isolated study and it is not clear how widespread such a phenomenon is. Nonetheless, MP exposure was found to aggravate asthma symptoms by increasing mucus production and inflammatory cell infiltration, as well as altering the expression of genes involved in programmed cell death, cellular stress response, and immune response in humans [90].

Microplastics can be inhaled and present in mammalian lung tissues which is very dangerous for animals. Li et al. [91] collected specimens from the lungs of domestic pigs and fetal pigs that died during vaginal birth. The samples were examined with polarized light microscopy and the Agilent 8700 LDIR chemical imaging system (LDIR). The polarized fluorescent microscope survey of domestic pig lungs revealed an average of 12 MP particles/g, which was higher than the 6 particles/g found in fetal pig lungs with sizes ranging from 115.14 mm to 1370.43 mm. The MP particles observed were all fiber shaped. LDIR analysis detected an average of 180 particles/g of domestic pig lungs with sizes ranging from 20.34 mm to 916.36 mm, which was twice the number of MPs found in fetal pig lungs. Polyamide (PA) was found to be the most common polymer in domestic pig lungs (46.11%), while polycarbonate (PC) was found to be the most common polymer in fetal pig lungs (32.99%). The presence of MPs was confirmed in the lung tissue of both domestic and fetal pigs in the natural environment, but the main characteristics differed. They hypothesized that the air and placenta were significant sources of exposure. The health risk posed by MPs through inhalation exposure should be considered. If the placental transfer of MPs can be confirmed in subsequent studies, it suggests fetuses may be exposed to MPs within the womb, which may even cause transgenerational effects on species.

Table 2 summarizes the works considered within this manuscript. The table provides a synopsis of current work in the field. While some innovative and provocative studies have been completed laying the groundwork for an understanding of microplastics impact on wildlife, much more research is needed. Follow-up studies should be encouraged to confirm and expand upon these seminal works. Section 4 below considers these gaps in more detail.

## 4. Conclusions & Future Research

**Figure 12:**
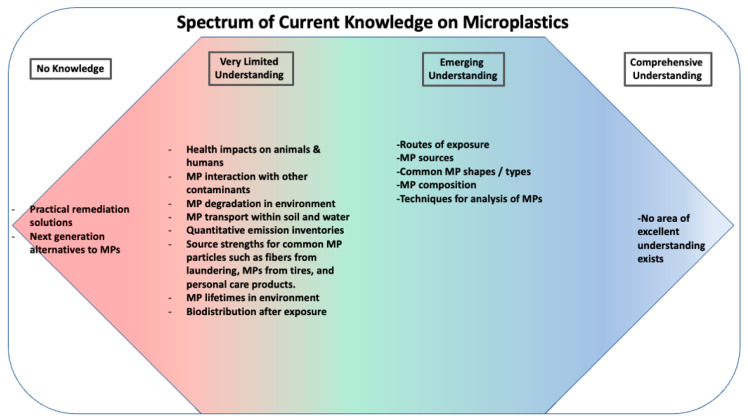
Spectrum of current knowledge regarding microplastics.

In conclusion, this manuscript outlines the prevalence of microplastics in wildlife and the negative impacts on their health and behavior. These findings highlight the urgent need for further research on the long-term effects of microplastics on wildlife and the development of effective management strategies for microplastics. Figure 12 reports the current state of knowledge regarding microplastics, their fate, and their impacts. At present, the understanding of microplastics is only beginning to develop. No topical area regarding microplastics and their impacts could be characterized as comprehensively understood. Recent work has begun to consider and identify the main routes of human and animal exposure to microplastics, sources of microplastics, their shapes, and common compositions. However, developing an understanding of the effect of microplastics on animal and human health and wellness may take decades of research efforts. In addition, developing well-constrained quantitative models for microplastic abundance in terrestrial sinks is of crucial importance. To accomplish this, science must develop a better understanding of microplastic removal mechanisms and rates as well as models for the generation rate of MPs. Research is sorely needed to understand the biodistribution of MPs—particularly as a function of size. From research on nanoparticles, it is well known that particles of nanoscale are typically integrated into and removed from organisms in very different ways compared to larger particles. This may be the case for nanoplastics, but the premise must be confirmed through well controlled research. The potential for biomagnification through the food chain must also be addressed.

In the laboratory, measurement methods for microplastics must be improved and standard reference materials developed [92]. Additionally, the development and implementation of long-term policy strategies for the reduction of plastic waste, such as increased recycling and the use of biodegradable alternatives, should be explored as a means of mitigating the negative impacts of microplastics on wildlife. At present no global remediation or microplastic reduction strategy has emerged. In addition, debate and discussion of the topic is not common among the world’s governing bodies. Developing a control strategy will prove challenging given the massive impact that synthetic polymers have on human lives and economies.

In summary, microplastic pollution is an emerging threat to human and animal well-being and further research attention should be focused on the topic. Specifically:

- Science must unravel the health impacts of MPs—especially as a function of size
- We need an improved understanding of the routes of human and animal exposure and resultant patterns of biodistribution
- A more comprehensive understanding of how MPs interact with other contaminants must be developed
- A better understanding of how MPs degrade in the environment should be developed
- Laboratories need improved and standardized methods to detect and quantify MPs
- Standard reference materials must be developed and made available to the research community
- Environmental impact assessments of emerging new polymer composites should be developed
- The world’s governing bodies should promote the development and debate of remediation solutions

Overall, the issue of microplastics in wildlife is a complex and pressing concern that requires further investigation, scientific funding, and action. By continuing to study the effects of microplastics and implementing effective management strategies, we can work towards protecting the health and well-being of wildlife and preserving their habitats for future generations.

## Figures and Tables

**Table 1:** Comparison of techniques for microplastics analysis.

Method	Advantages	Limits
Scanning Electron Microscopy (SEM)		
At the sample surface, a powerful electron beam is scanned. An electron beam’s scattering from the sample allows the images to be created.	This technique provides a high-resolution image of the sample (0.5 nm resolution).	The samples must be prepared for observation; type of polymer cannot be identified; high cost of instrument acquisition; tedious analysis
Raman Spectroscopy		
The target is exposed to laser radiation and inelastic scattering occurs. The chemical makeup of samples may be determined using the frequency shift between two beams.	Reliable method for identifying microplastics; can detect microplastics with a size to 1 µm; nondestructive; can analyze solutions and tolerates presence of water	The sample must be properly prepared to isolate MPs of interest; Raman spectroscopy can be affected by additives, dyes, or impurities, sample fluorescence background; acquisition of data can be time-consuming; expensive equipment required; interpreting data may be difficult without standards
Fourier-transform infrared spectroscopy (FTIR)		
Infrared light is used to illuminate samples, and absorbance or transmission spectra are then compared to those of known samples in libraries.	Dependable; nondestructive; micro-FTIR can study particles down to 20 µm in size; can determine the MP’s composition; ability to map the surface of a large sample.	When the target particles are smaller than 20 µm, the accuracy will decrease; expensive equipment; samples need to be pretreated/purified to remove matrix interferents; detection spatial resolution limited by wavelength of radiation to tens of microns at best; water cannot be present
Optical microscope		
Sample prepared and identified directly under the optical microscope	Rapid screening possible; lowest cost; ability to detect the size, shape, and color of MPs; easy to identify nonplastic particles when recognized	Lack of qualitative chemical identification; the potential to mistake polymer for inorganic materials; optical microscope may miss tiny particles.

**Table 2:** Summary of studies involving MPs and wildlife.

Species Affected	Key Results	Reference
Oncorhynchus mykiss Rainbow trout, *Sparus aurata* Gilthead seabream *Linnaeus*, and *Dicentrarchus labrax*	50–63% of fish have MPs in gastrointestinal tract >80% fibers of polyethylene, polyester, polyamide	[71]
Marine Copepod (*Tigriopus japonicus*)	decrease in fecundity in Copepod was observed	[72]
Various wild-caught fish	35.5% of fish had at least one piece of microplastic in their gastrointestinal tract. South Australia had the highest percentage of fish with plastic (49%) and Tasmania the lowest (20%). Mostly polyolefin	[73]
Zebrafish	Total food intake was reduced by 64.3–69.6% after exposure to MPs	[74]
Mussels	Mussels exposed to MPs bioaccumulated many PAHs	[75]
*Amphioctopus fangsiao*	Markers of oxidative stress were affected by exposure to MPs	[76]
Zebrafish	300–500 MP items found in liver—dose dependent, presence of MP affects absorption of chlorpyrifos	[77]
Shellfish	protozoan parasites were incubated with microplastic particles. Parasites accumulated on the surfaces of microplastics over time.	[78]
Flounders, cod, seabirds, otters, and seal	MPs present in liver, muscle, and intestines—in 2/3 of cod. Seven of the eight animals had MP in their intestines or stomach walls.	[79]
Fish larvae (*Cyprinus carpio*)	Larvae treated with 45.55 g, 91.1 g, and 136.65 g of microplastics per liter of water, indicators of oxidative chemistry affected	[80]
Barn owl (*Tyto alba*)	Found 33% diet pellets were containing MPs, mainly microfibers (88.2%)	[81]
Chicken embryos	Polystyrene particles at 1–1000 mg per liter of water administered to 12-day-old chicken embryos caused irregular myocardial tissue arrangement and altered biomarkers of stress.	[82]
Quail (*Coturnix Coturnix japonica*)	Fed MPs for 9 days and displayed significant reduction in body biomass in the MP-ingested group and total reactive oxygen species (ROS) levels in muscle and liver in quails are higher	[83]
Shearwaters (*Ardenna carneipes*)	Found significant effects of inflammation in inferior regions of the proventriculus of the birds and oedema, erythema (redness) and the loss of tissue structure on the epithelial surface of the proventriculus.	[84]
Little-black cormorant (*Phalacrocorax sulcirostris*)	MPs found were mostly films (75%), followed by fiber (18.75%), and fragments (6.3%) in type. Sizes of the microplastic was mostly (68.7%) between 100–1000 micrometer major dimension. The color of the microplastics found were transparent (56.2%), followed by red (18.7%), black (12.5), yellow, and blue (6.2% each).	[85]
Arctic Glaucous gull (*Larus hyperboreas*)	Authors found microplastics occurred in 14.3% of the birds’ intestines. MPs sampled were mostly polypropylene and polystyrene.	[86]
Blackbirds and Song Thrushes	No seasonal differences or age-related differences in microplastic ingestion in either species was observed.	[87]
Chickens	Interaction between gut microbiota and metabolites may be one of the mechanisms by which MPs exert their negative effects	[88]
Mice	MP exposure was found to aggravate asthma symptoms by increasing mucus production and inflammatory cell infiltration, as well as altering the expression of genes involved in programmed cell death, cellular stress response	[89]
Swine	Polyamide (PA) was found to be the most common polymer in domestic pig lungs (46%), while polycarbonate (PC) was found to be the most common polymer in fetal pig lungs (33%). The presence of MPs was confirmed in the lung tissue of both domestic and fetal pigs	[91]

## Data Availability

This work reports data from existing literature. No new data has been generated.
